# A Perfusion Bioreactor for Longitudinal Monitoring of Bioengineered Liver Constructs

**DOI:** 10.3390/nano11020275

**Published:** 2021-01-21

**Authors:** Lisa Sassi, Omolola Ajayi, Sara Campinoti, Dipa Natarajan, Claire McQuitty, Riccardo Rayan Siena, Sara Mantero, Paolo De Coppi, Alessandro F. Pellegata, Shilpa Chokshi, Luca Urbani

**Affiliations:** 1 Institute of Hepatology, Foundation for Liver Research, London SE5 9NT, UK; lisa.sassi@kcl.ac.uk (L.S.); l.ajayi@researchinliver.org.uk (O.A.); s.campinoti@researchinliver.org.uk (S.C.); dipa.natarajan@researchinliver.org.uk (D.N.); claire.mcquitty@researchinliver.org.uk (C.M.); rayan.siena@outlook.it (R.R.S.); s.chokshi@researchinliver.org.uk (S.C.); 2 Faculty of Life Sciences & Medicine, King’s College London, London WC2R 2LS, UK; 3 Department of Chemistry, Materials and Chemical Engineering “Giulio Natta”, Politecnico di Milan, 20133 Milan, Italy; sara.mantero@polimi.it (S.M.); a.pellegata@ucl.ac.uk (A.F.P.); 4 Stem Cells and Regenerative Medicine and Biomedical Research Center, NIHR, Great Ormond Street Institute of Child Health, UCL, London WC1N 1EH, UK; p.decoppi@ucl.ac.uk; 5 Specialist Neonatal and Pediatric Surgery, Great Ormond Street Hospital, London WC1N 3JH, UK

**Keywords:** bioreactor, tissue engineering, decellularization, liver, extracellular matrix, bioluminescence

## Abstract

In the field of in vitro liver disease models, decellularised organ scaffolds maintain the original biomechanical and biological properties of the extracellular matrix and are established supports for in vitro cell culture. However, tissue engineering approaches based on whole organ decellularized scaffolds are hampered by the scarcity of appropriate bioreactors that provide controlled 3D culture conditions. Novel specific bioreactors are needed to support long-term culture of bioengineered constructs allowing non-invasive longitudinal monitoring. Here, we designed and validated a specific bioreactor for long-term 3D culture of whole liver constructs. Whole liver scaffolds were generated by perfusion decellularisation of rat livers. Scaffolds were seeded with Luc^+^HepG2 and primary human hepatocytes and cultured in static or dynamic conditions using the custom-made bioreactor. The bioreactor included a syringe pump, for continuous unidirectional flow, and a circuit built to allow non-invasive monitoring of culture parameters and media sampling. The bioreactor allowed non-invasive analysis of cell viability, distribution, and function of Luc^+^HepG2-bioengineered livers cultured for up to 11 days. Constructs cultured in dynamic conditions in the bioreactor showed significantly higher cell viability, measured with bioluminescence, distribution, and functionality (determined by albumin production and expression of CYP enzymes) in comparison to static culture conditions. Finally, our bioreactor supports primary human hepatocyte viability and function for up to 30 days, when seeded in the whole liver scaffolds. Overall, our novel bioreactor is capable of supporting cell survival and metabolism and is suitable for liver tissue engineering for the development of 3D liver disease models.

## 1. Introduction

Liver tissue engineering is emerging as a suitable tool to facilitate the unmet need for in vitro liver models with physiological features of the native organ niche. Bioengineered liver constructs could form robust models to investigate cell metabolism, pathological mechanisms and perform drug screening and toxicity assay. Assays based on 2D cellular monolayers are not suitable to mimic the natural behaviours of hepatic cells in response to stimuli [1,2], since the 2D condition does not provide the hepatic architecture, biochemical gradients, cell-cell communication and cell-extracellular matrix (ECM) interaction. Mechanical stress generated by the stiffness of a petri dish affects the hepatic cell behaviour, by inducing phenotype trans-differentiation [3]. Moreover, consistent and reliable isolation and expansion of primary human hepatocytes still represents a challenge for therapeutic transplantation and laboratory research: in the absence of a 3D environment, hepatocytes rapidly dedifferentiate and down-regulate synthesis of metabolic enzymes within 24 h in culture [4]. Bioengineered liver tissue represents a valid strategy in recapitulating the hepatic microenvironment despite the intrinsic technical challenges in engineering such a complex organ. The hepatic architecture needs to be reproduced in vitro since it plays a crucial role in promoting cell communication and functions: metabolic activity of the hepatocytes, indeed, changes spatially along the sinusoids, depending on gradients of oxygen and ECM composition (liver zonation) [5,6]. Another challenge is the selection of appropriate biomaterials for cell scaffolding tailored to guarantee an appropriate 3D microenvironment. Decellularized scaffolds maintain biochemical and mechanical properties of the original tissue, guiding tissue regrowth according to the so-called contact-guidance theory, for which the cell behaviour is strongly influenced by the geometrical patterns, architecture and surface topography of the scaffold. Thickness of the construct can be an issue as scaffolds of clinically relevant size often results in the development of necrotic regions due to a lack of nutrient transport and oxygen diffusion [7]. Based on these complex requirements, bioreactors have the potential to revolutionize the standard culture procedure and represent a key tool in overcoming the challenges described in engineering liver tissue constructs. Bioreactors provide a suitable environment for the development of biological systems, under tightly controlled conditions and close monitoring of the variables which are well known to affect cell behavior [8]. There is a long history of bioreactor use in cartilage and bone engineering, but robust methods to develop and use bioreactors for liver tissue are lacking.

In this study we have developed a novel bioreactor-based technology, allowing long-term in vitro culture of liver scaffolds and providing dynamic medium and gas supply in a 3D perfusion system. The technology is based on a closed, sterile chamber connected to a programmable syringe pump, which provides a constant flow through the scaffold, optimizing the mass transport and exchange and the delivery of oxygen and nutrients. This bioreactor is also designed to allow non-invasive monitoring of cellular and perfusion parameters during culture which may be very relevant for disease modelling and pre-clinical toxicology studies. 

## 2. Materials and Methods 

### 2.1. Bioreactor Design

**Figure 1:**
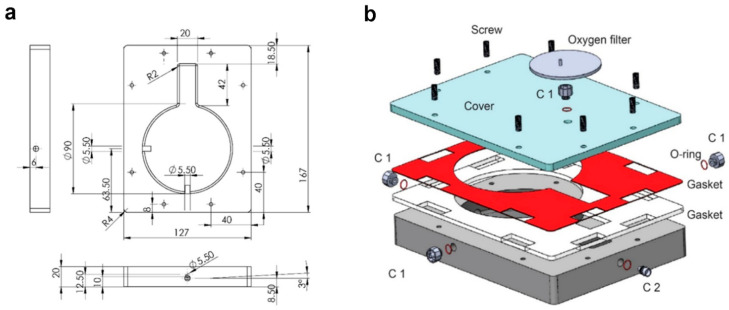
2D (**a**) and 3D (**b**) drawing of the chamber designed by computer-aided software (CAD, SolidWorks 2020) and manufactured using Computer Numerical Control machine. C1 and C2: poly-propylene connectors.

The bioreactor was designed by computer-aided software (CAD, SolidWorks 2020, Waltham, MA, USA) (Figure 1). The chamber was designed with a keyhole shape: a circular part (90 mm of diameter) for housing of the organ and a rectangular element (20 × 42 mm) for housing of cannulas and tubes. To facilitate the outflow and avoid fluid stagnation the internal flooring has been designed with an inclination of 3°. Sterility was ensured by a silicone double gasket. Nylon 6.6 (DirectPlastics, Sheffield, UK) was selected as suitable material, since it is autoclavable, biologically inert, shows chemical resistance and is available in black, making it compatible with bioluminescence non-invasive imaging. The chamber was manufactured using Computer Numerical Control machine (CNC, Roland, Shizuoka, Japan). The settings of the CNC machine are reported in Table S1.

### 2.2. Perfusion System

The system was based on a programmable syringe pump (World Precision Instruments R, Hitchin, UK) used as dispenser to perform a semi-continuous flow through the construct. To avoid presence of air, a Bubble Trap (Kinesis Scientific, Saint Neots, UK) linked to a Vacuum Assistance was implemented. The hydraulic circuit is characterized by an easy access three ways (Vygon, Swindon, UK) to perform media sampling. The system was assembled as shown in Figure 1b. 

### 2.3. Organ Harvesting

All surgical procedures and animal husbandry were carried out in accordance with the recommendations in the Animal (Scientific Procedures) Act 1986 and the local ethics committee. Wild type healthy adult Sprague-Dawley rats of 280–300 g were euthanized by CO_2_ inhalation, and the livers were isolated and harvested as previously described [9]. Briefly, the abdomen of the rat was sterilized with 70% Ethanol (EtOH; VWR, Leighton Buzzard, UK), the abdominal-pelvic cavity was exposed and the inferior vena cava (IVC) and portal vein (PV) were identified. The PV was cannulated with a 24G cannula (TERUMO, Fisher Scientific, Loughborough, UK) and the IVC was ligated with silk sutures (FST, Cambridge, UK). The whole liver was then released from the surrounding tissue. Sterile phosphate buffer saline (PBS) with 1 U/mL heparin (Sigma, Gillingham, Dorset, UK) was perfused to remove excess blood and check for leaks. 

### 2.4. Decellularization of Rat Liver

Decellularization was performed through the vasculature network directly after rat liver harvesting. The cannulated PV was connected to a peristaltic pump (iPumps, Pamington, UK) to perfuse solutions. A bubble trap (Kinesis Scientific) was exploited to ensure that no bubbles were perfused into the vasculature of the liver. The liver was perfused with MilliQ water for 18 h at a flow rate of 4.5 mL/min at room temperature, followed by 4% sodium deoxycholate (SDC; Sigma) for 5 h at 6.5 mL/min. Next, the rat liver was perfused at 6.5 mL/min with PBS for 1 h, then 3 h with 25 mg/L DNAse-I (Sigma, UK) in saline solution (0.15 M NaCl/10 mM CaCl_2_, Sigma), both pre-warmed and maintained at 37 °C. DNAse treatment was followed by perfusion of warm PBS for 1 h and finally PBS overnight at 1 mL/min at room temperature. Scaffolds were then sterilized by perfusion with 0.1% PAA/4% ethanol in milliQ water for 90 min, followed by 30 min of PBS with 1% penicillin-streptomycin (Sigma) and 50 ng/mL Primocin (Invitrogen, Waltham, MA, USA). Decellularized livers were finally gamma irradiated and stored at 4 °C in sterile PBS with 1% penicillin-streptomycin (Sigma) and 50 ng/mL primocin (Invitrogen) until use. 

### 2.5. HepG2 Cell Culture

HepG2 cells were cultured and transduced with pHIV-Luc ZSGreen based Lentivirus. The lentiviral transfer vector pHIV-LUC-ZsGreen was a gift from Dr. Bryan Welm [9] (Department of Surgery, University of Utah, purchased through Addgene, Inc., Watertown, MA, USA, plasmid #39196) (Addgene, Watertown, MA, USA). The ZSGreen and luciferase positive cells were then visualized with the substrate luciferin using an In Vivo Imaging System (IVIS). Lentivirus was added to cultures at multiplicities of infection in the range of 2–5, 100 µL for 10^5^ cells per well, and left for 36–48 h ensuring that the cells were transduced and the lentivirus had inactivated. Following trypsinization, FACS analysis was performed in order to both quantify the percentage of transduced cells, and to select eGFP+ cells. Transduced HepG2 cells were cultured using Dulbecco’s Modified Eagle Medium (DMEM) (Life Technologies, Renfrew, UK) supplemented of 10% fetal bovine serum (FBS; Life Technologies), 1% non-essential amino acids (NEAA; Sigma), 1% sodium pyruvate (100 mM; Life Technologies) and 1% L-glutamine (200 mM; Life Technologies).

### 2.6. HepG2 Seeding into Decellularized Rat Liver Scaffolds and Dynamic Perfusion Culture

The decellularized rat liver was primed with 9 mL of HepG2 media. A total of 50 × 10^6^ HepG2 were seeded in the rat scaffold. The seeding was carried out though 4 perfusion steps, 12.5 × 10^6^ HepG2 for each step with 30 min of rest between injections. Cells were delivered into the scaffold through the portal vein (PV) at 9 mL/min. After 24 h, the seeded construct was divided into two parts to obtain two scaffolds for either dynamic perfusion culture or static culture respectively, for 11 days. For dynamic perfusion 20 mL medium was pumped at 9 mL/min and subsequently withdrawn at the same speed using the automated syringe pump. The media were changed every 2 days and sampled every day from both 3D cultures.

### 2.7. Primary Human Hepatocytes Seeding 

Primary human hepatocytes were kindly donated by Lonza (Morristown, NJ, USA) and stored in liquid nitrogen until the day of the seeding. A total of 5 vials of primary human hepatocytes were thawed in Hepatocytes Thawing medium (Lonza) following manufacturer’s instructions. The average cell viability post thawing was 94.4%. Seeding was performed following the same protocol developed for HepG2 seeding in rat liver decellularized scaffold. A total of 50 million cells were seeded performing 5 perfusion steps through the portal vein (PV) at 4 mL/min in a volume of 6 mL (per perfusion step) of Hepatocytes Plating medium (Lonza). After 24 h, the seeded construct was divided into two parts to obtain two scaffolds for either dynamic perfusion culture (at 1 mL/min) or static culture respectively, for 30 days. Static and bioreactor 3D cultures were performed in Hepatocytes Incubation Medium composed of: William’s E Medium (no phenol red, Sigma), 5% FBS (Life Technologies), 1% penicillin-streptomycin (Sigma) and 50 ng/mL primocin (Invitrogen), 1% glutamine (Invitrogen), 15 mM HEPES pH7.4 (Invitrogen), 0.1 µM dexamethasone (Sigma), 1X insulin-transferrin-selenium (IST-G, Life Technologies), 10 mM nicotinamide (Sigma) and 50 ng/mL human recombinant EGF (Peprotech, London, UK). The medium was changed every 4 days and sampled every other day from both 3D cultures.

### 2.8. Bioluminescence Analysis

For analysis at IVIS (Perkin Elmer, Waltham, MA, USA), the chamber was perfused with luciferin (10 µg/mL; Cayman Chemicals, Ann Arbor, MI, USA). Images were acquired using the Lumina III In Vivo Imaging System (IVIS) and the Living Image 4.4 Software. The black bioreactor chamber enhanced the bioluminescence visualization. The software generates pseudo-coloured, scaled images overlaid on grey scale images, providing 2-dimensional localization of the source of light emission. All images were taken using stage E, with automated aperture setting, exposure time of 5 min and small binning (resolution). Regions of interest (ROIs) were selected using shape drawing tools and the light emission within the ROI was quantified as average radiance ([photons/sec/cm^2^/steradiant]). ROI were kept constant between subjects within each experiment. Scaffolds were imaged every day or alternate days for bioluminescence. 

### 2.9. Histology

#### 2.9.1. Hematoxylin & Eosin

Tissue cryosections were prepared with a cryostat (Bright from Leica Biosystems, Wetzlar, Germany) for H&E staining. Sections of 10–12 µm were rehydrated in PBS at 37 °C and then left for 5 min in hematoxylin, 1 min in alcoholic acid and 5 min in eosin. Sections were then rehydrated (70%, 90% 100% EtOH, each phase for 1 min) and mounted with DPX after having immersed them in Histoclear (National Diagnostics, SLS, Nottingham, UK). 

#### 2.9.2. Immunofluorescence

Scaffold segments were fixed with 4% paraformaldehyde (PFA) for 1 h at RT. Tissue pieces were washed in PBS twice and left overnight at 4 °C in 30% sucrose (Sigma) in PBS. Subsequently, samples were embedded in 15% sucrose/7.5% gelatine in PBS, frozen in ice cold isopentane and sectioned at 10 µm with a cryostat (Bright). Sections were rehydrated in PBS at 37 °C and permeabilized in 0.1% triton in PBS (PBS-T) for 10 min followed by blocking using an appropriate blocking serum. Primary antibodies used, their concentration and blocking serum are listed in Table S2. Slides were incubated with primary antibodies overnight at 4 °C. Sections were then incubated with AlexaFluor-488 or AlexaFluor-568 conjugated secondary antibodies (dilution 1:500; Life Technologies) at RT for 1 h. Nuclei were counterstained with DAPI (dilution 1:1000, Life Technologies). 

### 2.10. Albumin and Urea Quantification

To test the functionality of the hepatocytes and hepatoma cultures, aliquots of media was collected every 2 days from both static and dynamic perfusion cultures. Albumin content in the medium was measured using the Human Albumin ELISA kit (E88-129, Bethyl Laboratories, Inc. Montgomery, TX, USA) following manufacturer’s instructions. Culture media was diluted 1:2 before assaying.

Urea content was measured using colorimetric Urea assay kit (ab83362, Abcam, Cambridge, UK) following manufacturer’s instructions. Both static and dynamic perfusion culture media was diluted 1:32 before assaying. 

### 2.11. RNA Isolation and RT-qPCR

2D cultured HepG2 and primary human hepatocytes and 3D seeded scaffolds were digested with Trizol TRI Reagent (Sigma-Aldrich, St Louis, MO, USA) and RNA was extracted following the manufacturer’s instructions. Precipitated and dried RNA was re-suspended in nuclease free water (Qiagen, Hilden, Germany). RNA concentration was measured using Nanodrop1000 (ThermoScientific, Waltham, MA, USA). RNA was converted into cDNA using GoScript™ Reverse Transcriptase kit (Promega, Southampton, UK) according to the manufacturer’s protocol. cDNA concentration was adjusted to 10 ng/µL. Quantitative (q)PCR was performed on 25 ng of cDNA using PCR master mix (PrecisionPLUS-R—Primerdesign Ltd. Chandler’s Ford, UK) with low-ROX and Taqman qPCR probes (Integrated DNA Technology, Coralville, Ia, USA. List of probes in Supplementary Table S2) in MicroAmp Fast Optical 96 well Reaction Plates (Starlab, Milton Keynes, UK) using the ABI7500 Real-Time PCR System (Applied Biosystems, Foster City, CA, USA). Target genes are reported in Table S3.

### 2.12. DNA Quantification

To determine whether decellularisation had effectively removed native DNA, the total DNA was isolated from fresh liver and decellularised liver samples using DNeasy Blood and Tissue kit (Qiagen) as per the manufacturer’s instructions. Purified DNA was quantified using Nanodrop (ThermoScientific, Waltham, MA, USA). 

### 2.13. Mycoplasma Test

To test the sterility of the culture, mycoplasma contamination was periodically tested with a commercial detection kit, MycoProbe (R&D Systems, Minneapolis, MN, USA) following the manufacturer’s instructions. Briefly, media or supernatants from cell cultures or scaffold storage cultures were incubated in specialized microplates coated with biotin-labelled capture oligonucleotide probes and digoxigenin-labelled detection probes. The presence of 16S ribosomal RNA of the eight most common mycoplasma contaminants in the samples would lead to a hybridization with the probes. The hybridization solutions were then transferred to a streptavidin-coated microplate, with any hybridized RNA being captured, the addition of an anti-digoxigenin alkaline phosphatase conjugate and a substrate solution would develop colour with absorbance at 490 nm spectrophotometrically. O.D. values were then compared to the positive control; samples were free of mycoplasma contamination when calculated O.D. value < 0.10. 

### 2.14. Endotoxin Quantification

Endotoxin contamination was periodically tested using a Cloud-Clone Corp kit (SEB526Ge, Katy, TX, USA) ELISA kit for lipopolysaccharide (LPS). Cell culture supernatant was stored at −20 prior to testing and assayed neat. 

### 2.15. Collagen Quantification

Total collagen content of fresh and decellularized rat liver samples was quantified with Collagen Assay Kit (QuickZyme, Biosciences, Leiden, The Netherlands) as per manufacturer’s instructions. The concentration was evaluated via spectrophotometry measurements of hydrolysed samples at 555 nm via interpolation with a standard curve produced with known collagen concentrations. 

### 2.16. Statistical Analysis

Statistical analysis was carried out using Graphpad Prism Software (Groningen, The Netherlands) and Student’s t-test was used to compare between two groups. Two-tailed *p*-values of data were assessed using Student’s *t*-test. In all figures, statistical significance is expressed as *** *p* < 0.001, ** *p* < 0.01 and * *p* < 0.05. Quantitative data was expressed as mean ± standard deviation (SD) and graphs as mean ± standard error (SEM). 

## 3. Results

**Figure 2:**
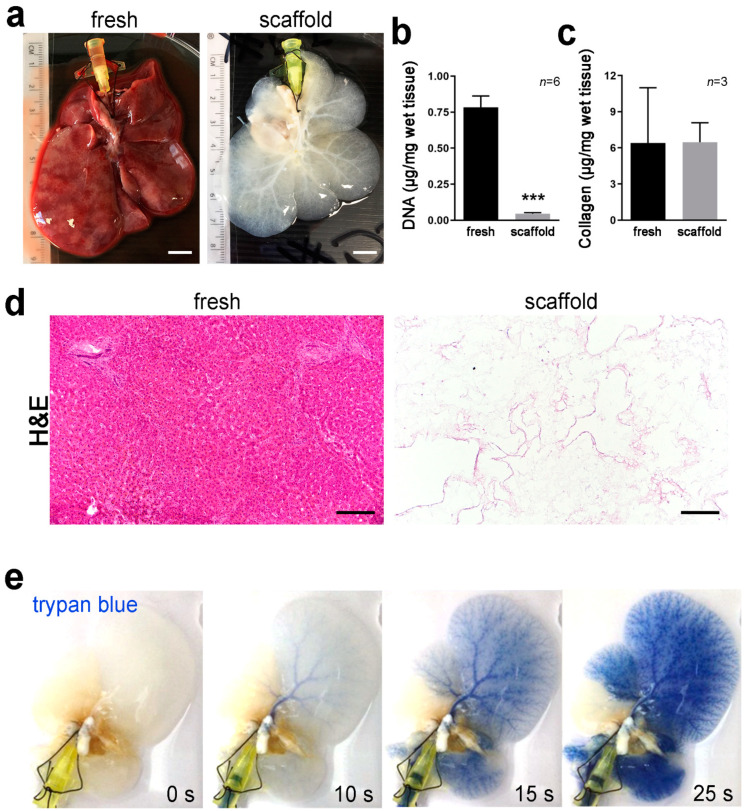
Decellularization of rat whole liver. (**a**). Rat liver with cannulated portal vein before (left) and after (right) detergent-enzymatic perfusion decellularization showing change in tissue colour during the process. Scale bar: 2 cm. (**b**). DNA quantification in segments of fresh liver tissue and decellularized liver scaffolds. *** = *p* < 0.001 *t*-test. (**c**). Collagen quantification in segments of fresh liver tissue and decellularized liver scaffolds. (**d**). H&E staining of fresh and decellularized rat liver tissue showing absence of nuclei and cytoplasm. Scale bar: 200 µm. (**e**). Snapshots recorded at 0, 10, 15 and 20 s of trypan blue dye perfusion through the portal vein of a decellularized scaffold to highlight intact vasculature tree.

Decellularized rat livers were obtained using an established detergent-enzymatic treatment [10]. Perfusion of decellularization reagents was performed through the cannulated portal vein (PV). The process eliminated the cellular compartment preserving the ECM, resulting in a translucent scaffold with visible vasculature (Figure 2a). DNA quantification in tissue segments confirmed elimination of the cells upon decellularization (Figure 2b), while collagen, the main ECM protein in the liver, was preserved (Figure 2c). H&E staining highlighted absence of nuclei and cytoplasm in the decellularized scaffolds and preservation of the overall matrix structure (Figure 2d). Trypan blue dye perfused into the decellularized scaffolds through the PV allowed for clear visualization of the intact vascular network, showing no leakage of dye (Figure 2e).

**Figure 3:**
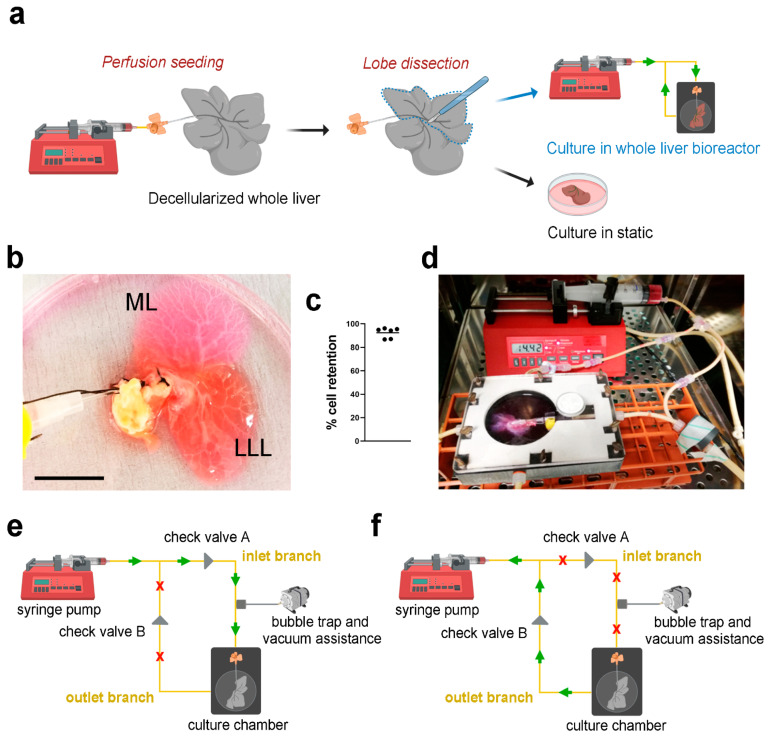
The perfusion system of the bioreactor. (**a**). Schematic of perfusion seeding into decellularized whole liver scaffolds, dissection and static or perfusion culture conditions. The scaffolds were seeded via the canula. Upon seeding, a lobe was dissected and cultured in static conditions while the other lobes were connected to the bioreactor circuit via the canula and cultured in dynamic perfusion condition. (**b**). Representative image of a decellularized scaffold seeded with Luc^+^HepG2. Scale bar: 2 cm. (**c**). Percentage of cell retention in the scaffolds upon seeding. (**d**). Photo of the bioreactor system assembled with the chamber containing a decellularized scaffold primed with culture medium before seeding. The pump is connected to the chamber through two branches, the inlet branch and the outlet one. (**e**). Syringe pump set to “pumping” mode: medium is pushed through the inlet branch and diffused through the vasculature network. (**f**). Syringe pump set to “withdrawing” mode: medium is withdrawn through the outlet branch from the chamber, returning to the syringe. ML: median lobe; LLL: lateral left lobe.

HepG2 cells transduced with pHIV-Luc-ZSGreen based lentivirus (Luc^+^HepG2) were perfused into the decellularized scaffolds through the PV using a syringe pump (Figure 3a), showing evident infiltration of cells in both median lobe (ML) and lateral left lobe (LLL) (Figure 3b). Cell retention into the scaffolds was evaluated by counting cells in the perfused media surrounding the liver scaffolds after seeding. Almost 100% of the cells perfused were retained inside the scaffolds (Figure 3c). The repopulated scaffolds were then separated placing the ML in static culture and the LLL into bioreactor perfusion culture, both cultures were incubated for up to 11 days (Figure 3a,d). Perfusion culture was obtained through the use of a closed-loop circuit, where the pump was connected to the chamber through two branches, the inlet branch and the outlet branch. When in “pumping” mode, the syringe pump pushed media through the inlet branch connected to the cannulated PV, diffusing through the vasculature network (Figure 3e). Media was then removed from the chamber back into the syringe once the pump was in “withdrawing” mode, via the outlet branch (Figure 3f). The presence of two check valves positioned at the two branches allowed correct directional functioning of the system: their role was to direct the fluid, allowing the pumping phase only through the inlet branch and the withdrawing phase through the outlet one.

**Figure 4:**
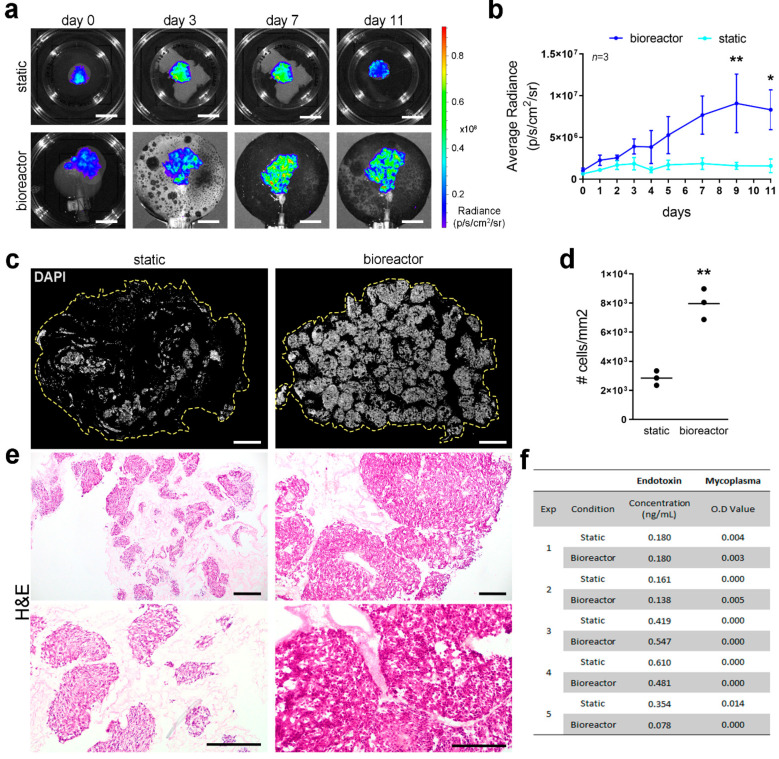
Cell viability, distribution, and density in 3D cultures. (**a**). Representative bioluminescence images at different time points of seeded ML and LLL from the same decellularized liver cultured in static and perfusion bioreactor conditions, respectively. Scale bar: 2 cm. (**b**). Bioluminescence readings up to 11 days of culture (*n* = 3). * = *p* < 0.05; ** = *p* < 0.01 2-way ANOVA, Bonferroni’s multiple comparison’s test. (**c**). Representative images for staining with DAPI (grey) to show distribution of nuclei in cross-sections. Scale bar: 200 µm. (**d**). Number of cells per area determined in images from DAPI staining (**e**). Representative images of H&E staining of scaffolds cultured in static condition or in the bioreactor. Scale bar: 200 µm. (**f**). Mycoplasma and endotoxin concentration in the media at day 11 of static or bioreactor cultures in 5 different experiments.

Bioluminescence imaging was used for longitudinal assessment of cell distribution and viability by perfusing luciferin via the bioreactor or directly into the culture plate for static cultures. Bioluminescence clearly showed initial cell distribution in the proximal area of both static and perfused scaffolds, with comparable levels of bioluminescence detected in both conditions, indicating a similar number of viable cells in the two scaffolds (Figure 4a). A steady increase in cell viability and an overall homogenous distribution of cells was detected during culture, with significant higher cell viability in scaffolds cultured in perfusion conditions compared to static cultures, reported as average radiance (Figure 4b). Scaffolds repopulated with Luc^+^HepG2 cells were embedded for cryosectioning or snap frozen at the end of the culture period to further assess cell density and distribution through DNA quantification and stainings. Although cells appeared to have reached different areas of the scaffolds under both culture conditions, a clear improvement in cell growth and distribution was shown in perfusion cultures compared to static conditions (Figure 4c–e). Cell density was significantly higher in bioreactor cultured scaffolds compared to static conditions as indicated by the higher number of cells per area (Figure 4d) and as appreciable by H&E staining (Figure 4e). Endotoxin levels and mycoplasma contamination were quantified in the circulating media or static media after 11 days of culture and both values resulted below the thresholds indicated by the kit’s manufacturers and by regulatory agencies. No difference in endotoxin and mycoplasma levels was detected between bioreactor and static culture, demonstrating that the bioreactor maintained the same level of sterility in respect to conventional static cultures (Figure 4f). 

**Figure 5:**
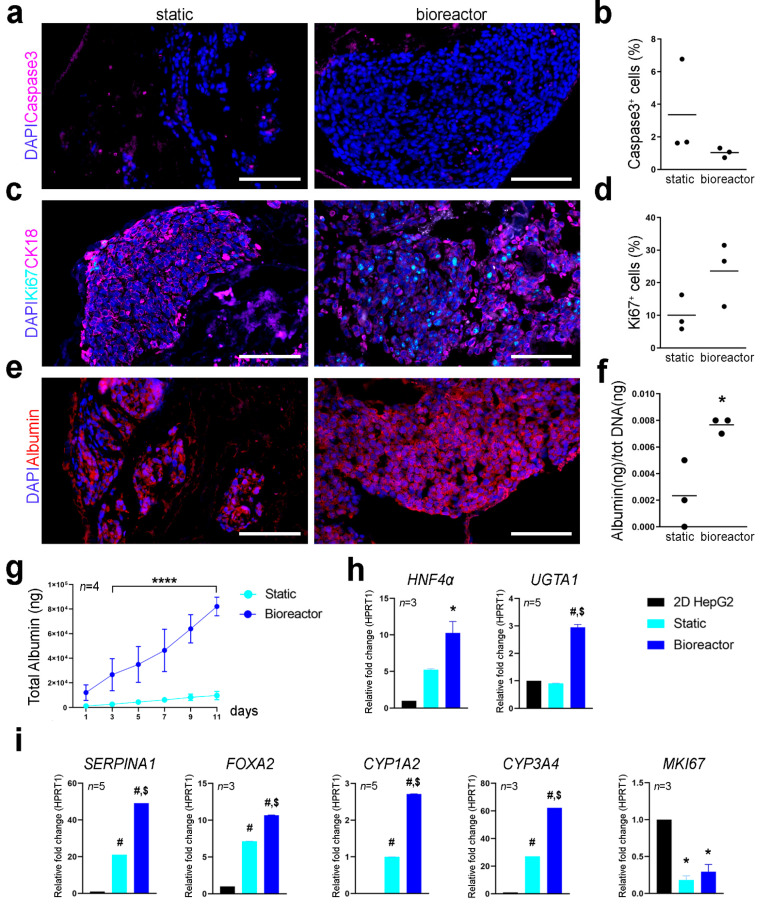
Characterization of scaffolds recellularized with Luc^+^HepG2 cells and cultured with or without bioreactor. (**a**,**c**,**e**). Immunofluorescent staining for caspase-3 (magenta) (**a**); Ki67 (cyan) and CK18 (magenta) (**c**); albumin (red) (**e**); nuclei were counterstained with DAPI (blue). (**b**,**d**) Quantification of caspase-3^+^ cells (**b**) and Ki67^+^ cells (**d**) over DAPI count per area, represented as mean ± SD. Scale bar: 100 µm. (**f**). Albumin quantification (in ng) in the media of static and bioreactor cultures at day 11. Nanograms of albumin were normalized against total DNA (ng) in scaffolds after static and bioreactor culture conditions respectively. (**g**). Albumin quantification represented as albumin production per day (in µg), **** = *p* < 0.0001 Two-way ANOVA. (**h**,**i**). Gene expression profile of HepG2 cells in 2D conditions and in static and perfusion 3D cultures. qPCR was performed on 8 samples of scaffolds in static or dynamic perfusion culture for a total of 4 independent experiments. Data is shown as relative fold change in respect to the reference gene *HPRT1* as mean +/− SEM. Gene expression of HepG2 cells in 2D conditions was used as reference (1) for all genes with exception of CYP1A2, of which transcripts were not detected in HepG2 in 2D samples. * = *p* < 0.05; # = *p* < 0.0001 *t*-test vs. 2D cultures. $ = *p* < 0.0001 static vs. bioreactor culture.

Cell proliferation and apoptotic rate were assessed using immunofluorescence for Ki67 and caspase-3 on cryosections. Cell apoptosis and proliferation at day 11 seemed comparable between the two culture conditions with no significant difference in the percentage of caspase-3^+^ and Ki67^+^ cells (Figure 5a–d). Expression pattern of CK18 was also comparable between static and bioreactor perfusion culture (Figure 5c). Albumin was expressed by Luc^+^HepG2 cells cultivated in static and perfusion culture conditions as shown by immunofluorescence analysis, although some cells were negative for albumin expression in static cultures (Figure 5e). Difference in the metabolic activity of cells cultured in 3D scaffolds with or without the bioreactor was determined through albumin quantification and qPCR of hepatocyte specific factors. Bioreactor cultured constructs showed higher amount of albumin produced at day 11 of culture and a higher total cumulative albumin throughout the culture period compared to static culture (Figure 5f,g), suggesting enhanced hepatic cell metabolic function when cultured in the bioreactor. Gene expression profiling of 3D cultured Luc^+^HepG2 cells was determined with qPCR. Transcription levels for hepatocyte nuclear factor 4-α (*HNF4α*), UDP-glucuronosyl- transferasy1-1 (*UGTA1*), *SERPINA1*, forkhead box A2 (*FOXA2*), cytochrome P450 family 1 subfamily A member 2 and family 3 subfamily A member 4 (*CYP1A2* and *CYP3A4*) and *MKI67* were analysed using *HPRT1* as reference gene and normalising gene expression on Luc^+^HepG2 cells cultured in conventional 2D culture conditions (Figure 5h,i). The *SERPINA1* gene, which encodes for hepatocyte’s serine protease inhibitor α-1-antitrypsin [11,12] resulted to be significantly upregulated in bioreactor cultures compared to static culture conditions and 2D cultured cells. Luc^+^HepG2 3D cultured in the bioreactor showed upregulation of *HNF4α* in respect to conventional 2D cultures. HepG2 cultured in standard 2D conditions do not express *CYP3A4* at high levels [13,14], however, this gene was upregulated in 3D cultured Luc^+^HepG2 cells, in particular in perfusion cultures. A similar upregulation was evident for other genes crucial for hepatocytes functions such as *FOXA2* and *CYP1A2*. As expected, *MKI67*, transcript of proliferation marker KI67, was downregulated in 3D conditions.

**Figure 6:**
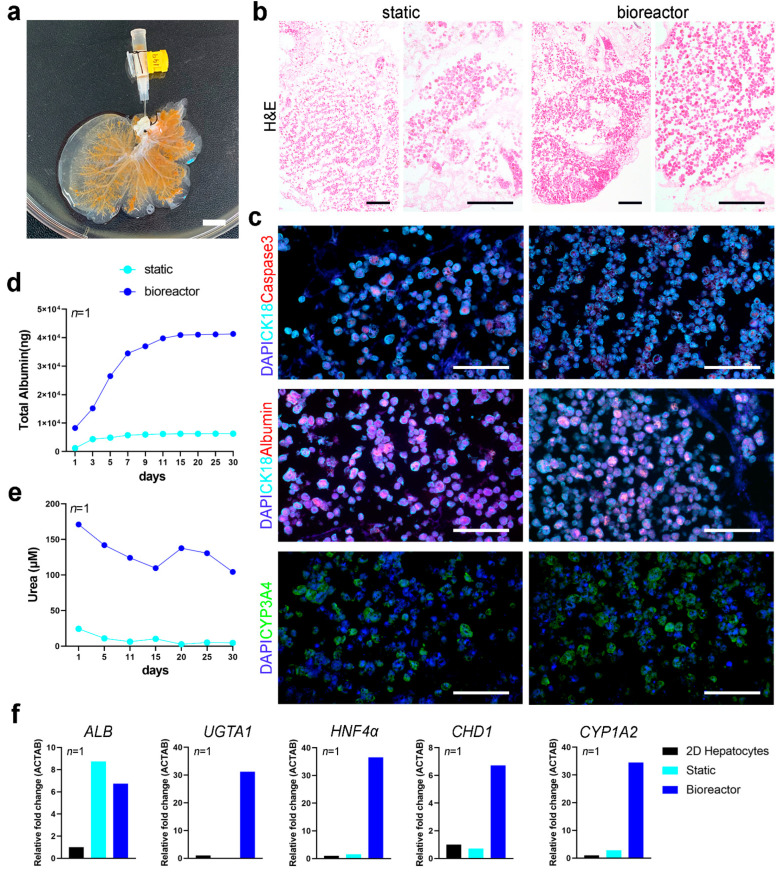
Characterization of scaffolds recellularized with primary human Hepatocytes and cultured with or without bioreactor for 30 days. (**a**). Representative image of a decellularized scaffold seeded with primary human Hepatocytes through the cannulated PV. Scale bar: 1 cm. (**b**). Representative images of H&E staining of scaffolds cultured in static condition or in the bioreactor for 30 days. Scale bar: 200 µm (**c**). Immunofluorescent staining of scaffolds repopulated with primary hepatocytes and cultured in static conditions or in bioreactor for 30 days. Top: staining for caspase-3 (red) and CK18 (cyan); middle: staining for albumin (red) and CK18 (cyan). Bottom: staining for CYP3A4 (green). Nuclei were counterstained with DAPI (blue). Scale bar: 100 µm. (**d**). Albumin quantification (total albumin in the media, in ng) over the 30 days of culture of the scaffold in static condition or in the bioreactor. (**e**). Urea quantification (total urea in the media, in µM) over the 30 days of culture of the scaffold in static condition or in the bioreactor (**f**). Gene expression profile of primary hepatocytes in 2D conditions and in static and perfusion 3D cultures. Data is shown as relative fold change in respect to the reference gene *ACTAB*. Gene expression of hepatocytes cells in 2D conditions was used as reference (*n* = 1).

To test whether the bioreactor could support the long-term culture of human primary cells, we performed a proof of principle experiment seeding a whole rat liver scaffold with primary human hepatocytes (Figure 6a). Lobes were then separated to place the ML in static culture and the LLL in perfusion culture in the bioreactor for up to 30 days. H&E staining of scaffolds at 30 days of culture showed higher repopulation in scaffolds in the bioreactor compared to static culture conditions (Figure 6b). Immunofluorescence staining showed that hepatocytes in scaffolds retained their expression of CK18 and had comparable levels of albumin expression, while fewer cells were positive for apoptotic marker caspase-3 in scaffolds cultured in the bioreactor for 30 days (Figure 6c). Immunostaining for CYP3A4 evidenced similar distribution of positive cells between static and bioreactor cultured hepatocytes, with relative higher expression in perfusion cultured constructs (Figure 6c). The cellular metabolic activity in 3D cultures was determined through albumin and urea quantification (as a surrogate for hepatocyte mediated ammonia detoxification) and qPCR of hepatocyte specific markers. Over the 30 days of culture, primary human hepatocytes in bioreactor culture produced higher amount of albumin than cells in 3D static culture conditions (Figure 6d). Hepatocytes cultured in the bioreactor also produced more urea throughout the 30 days of culture (Figure 6e). qPCR analysis showed that hepatocytes cultured in perfusion conditions upregulated functional genes, such as *UGTA1*, *HNF4α* and *CYP1A2*. *CDH1* gene, encoding for E-cadherin adhesion molecule, was also upregulated in hepatocytes in the scaffold cultured in the bioreactor, indicating a better cell-cell and cell-ECM adhesion (Figure 6f). Albumin (*ALB*) transcripts levels were comparable between static and bioreactor culture conditions. 

## 4. Discussion

In this study we have described a novel perfusion-based bioreactor technology to support the generation of whole-organ models. Our platform exploits the original hepatic vascular network as an effective route for seeding of hepatic cells, perfusion of nutrients and longitudinal monitoring of cell distribution, viability and function. The bioreactor fulfilled the most important ideal technical specifications, namely cytocompatibility, reliability, sterility, limited incumbrance, versatility, increase of nutrients and gas transport, biophysical stimulation, and automation. 

The 3D ECM-scaffold was generated by established perfusion decellularization of rat livers. The maintained 3D native architecture provided an ideal platform for hepatic cultures. The preservation of vasculature networks was one of the key advantages of this model together with the custom design of the bioreactor allowing whole organ upscale, longitudinal non-invasive analysis and extended culture capability. The physiological environment constituted by the hepatic ECM is a tissue-specific architecture of both structural and functional proteins maintained by a precisely regulated equilibrium between synthesis and degradation [15]. The ECM harbors a range of growth factors and other matrix-associated molecules which influence cellular activity [16]. ECM also provides cells with signals for polarization, adhesion, migration, proliferation, survival and differentiation [17]. ECM obtained by decellularization provides a characteristically appropriate environment to support hepatic cell repopulation and tissue functionality, and its use was maximised in our perfusion culture system to extend and maintain long-term cultures.

Liver disease represents one of the most important global human health issues, which has driven a prominent strive forward in liver regenerative medicine. To investigate physiological and pathological hepatic mechanisms, conventional 2D cultures and animal models present significant drawbacks [18,19,20,21]. Recently, several reports have been published on the use of decellularized liver ECM for the development of models for liver function and diseases [22,23,24,25]. However, the development of an efficient bioreactor-based culture system for 3D hepatic structures represent one of the major constraints of the field. A limiting factor in whole-liver tissue engineering has been that bioreactors usually play a marginal role, confined to a temporary support for the generation of the construct [26,27,28]. 

The bioreactor in this study was developed in Nylon 6.6, which is FDA-approved and inert, with high chemical resistance and is not susceptible to corrosion or release of cytotoxic products, events we confirmed were absent in our long-term culture experiments. The efficiency of cell seeding as well as monitoring cell fate during long-term culture is currently determined retrospectively by histological analyses, DNA quantification assay and metabolic activity analyses. These techniques provide information at a fixed time point and are limited by the requirement of termination of the experiment for analysis. In the case of long-term cultures, a reliable tool to monitor and track cells at different time points is crucial. Nylon 6.6 is compatible with the use of bioluminescence imaging (BLI) [9], representing an innovative feature not yet exploited in the field of bioengineered livers. BLI is a powerful tool utilised in our system which overcomes many of these drawbacks. The procedure for BLI is non-invasive, allowing for longitudinal monitor of cells in the bioreactor at different time points. The bioreactor has been successfully designed with a black nylon chamber to ensure enhanced BLI visualisation upon direct injection of luciferin inside the scaffold through the PV, and concomitantly sampling of culture media whilst limiting exposure to contaminants, maintaining sterility. The entire system fits into a standard incubator, allowing easy access to the control panel during culture, without the need for expensive and difficult adjunct modalities. This allows for simple assembly and use, changing of culture media and addition of chemical/toxic compound during culture by replacement of the syringe or using the three-way access point. The bioreactor is scalable and easy to use and could be applied to other whole-liver cultures or other bioengineered whole-organs such as pancreas and kidney. 

It is well-established that cells have a limited autonomy after a certain distance from a nutrient source, they can generally survive within an area of 1 mm away from a vessel [29]. This feature assumes paramount importance in the field of hepatic bioengineering because of the high rate of hepatocyte oxygen consumption; hepatic tissues should contain an extensive micro-vascular network to ensure constant nutrient supply and avoid any ischemic damage [30,31]. The perfusion within our system recreated a physiological-like delivery of nutrients, mimicking an environment where cells can reach optimal functionality, expansion and distribution. The flow rate selected for seeding and dynamic perfusion culture was 9 mL/min, the most conducive mechanical environment, closely emulating physiological blood flow through the PV of a rat liver [32]. Moreover, this flow rate has been previously reported to induce optimal HepG2 proliferation and tissue reorganization [26]. The correlation between fluid dynamic stimulation and cell engraftment, proliferation and behaviour in engineered tissues has yet to be fully determined and further studies are required to investigate the role of liquid flow in these systems. Our bioreactor technology could represent a suitable device to explore the crosstalk between these parameters. 

Hepatocyte or hepatoma cell lines represent a valid cell source in liver bioengineering. Due to their proliferation properties and metabolic prolife, HepG2 cells have been shown to provide a suitable alternative to primary hepatocytes, in pharmaceutical research, metabolism studies or sub-chronic to chronic hepatotoxicity studies [33], and in hepatic bioengineering with decellularized scaffolds [10,26,34]. Here we have reported a technology able to support culture and longitudinal analysis of HepG2-seeded scaffolds for up to 11 days, showing superior performances compared to static cultures. At 11 days, HepG2 cells were found repopulating almost all the scaffold area, with higher repopulation efficiency in dynamic perfusion condition in comparison to static. Furthermore, our system supported long-term survival and function of primary human hepatocytes for up to 30 days in proof-of-principle experiments. Cultivation of primary hepatocytes cells in liver scaffold employing a dynamic perfusion system has also been previously shown to improve cellular distribution within the scaffold and the oxygenation of the engineered construct [35]. Mazza et al. have shown increased functionality in 3D dynamic culture up to 10 days; here we support these data and we have implemented the analysis by cultivating primary human hepatocytes for a month; primary hepatocytes were found at high frequencies after prolonged in vitro culture, showing viability and expressing ontogenetic markers (i.e., CK18) and functional markers (i.e., albumin and CYP3A4). We showed that at gene and protein levels, both human primary hepatocytes and HepG2 cells were more functional in 3D bioreactor perfusion cultures than in static conditions, supporting the essential role of the bioreactor. Hepatic cell cultures in our perfusion bioreactor showed albumin secretion and urea production comparable to what has been previously shown by Robertson et al., 2018 in a similar rat liver model cultivated for 28 days with the use of a bioreactor system [24]. 

These results show that bioreactor-perfusion culture of complex bioengineered livers provides a more physiological environment that supports long-term culture of functional hepatocytes. This perfusion-based bioreactor could be easily used to culture scaffolds seeded with iPS-derived hepatocytes or hepato-biliary progenitors from liver organoids to evaluate whether a dynamic culture could support their survival, proliferation, and maturation. In this context, Wang and colleagues have shown advantages of ECM derived liver scaffold versus PLLA-collagen bioscaffolds in promoting specific hepatocyte marker expression and boosting the liver function of iPSCs [36]. Furthermore, in a recent report by de l’Hortet et al., the authors reported the biofabrication of genetically edited human liver tissue to mimic fatty liver disease starting from iPSC [37]. Liver organoids and iPSC are widening the possibilities to develop complex 3D models of disease and a perfusion bioreactor could extend/support these cultures, similarly to what recently shown in an in vitro whole-organ bioreactor grown artificial liver model (BALM), developed with iPSCs-derived hepatocyte-like cells [38]. Historically, models incorporating peristaltic pumps produce periodic compression of tubing, making the system unsuitable for the perfusion of circulating cells through the tissue or organ. Our system has the potential to incorporate perfusion of immune cells creating an immunocompetent liver model, highly sought in advanced liver disease modelling. This addition would permit intricate investigations of interactions between immune cells and hepatic cells in addition to hepatic cell-ECM interactions, recapitulating the complex liver microenvironment and inflammation-mediated pathology which is a central ‘tenet’ in the progression of chronic liver disease.

An important drawback of decellularisation is the loss of the organ endothelial layer. In the absence of such cells, coagulation can be easily triggered upon in vivo transplantation of the tissue engineered constructs, when blood is exposed to the ECM. Furthermore, the role of liver endothelial cells in physiological and pathological condition is critical and has to be taken into account in an appropriate liver disease model [39]. For this reason, it is essential to develop strategies that can allow hemocompatibility and re-endothelialisation of the scaffolds, similarly to what has been adopted for other organs [40,41]. Our perfusion seeding and culture system via canulation and the use of a syringe pump would also be appropriate for the reconstruction of the natural liver vascular tree and this is an area that warrants further investigation. 

At present, bioreactors have been mainly exploited in clinical applications using human liver cells to support hepatic function in patients with acute liver failure [42,43]. Hollow-fibre bioreactors have been described as valuable tools to support the generation of small hepatic constructs as valid alternative for pharmacological studies [44,45]. These bioreactors are designed with the aim to maximise the delivery of nutrients and gas supply, but do not consider aspects crucial for liver function, for example the hepatic architecture. Bioartificial liver (BAL) support systems have the potential to provide temporary support to bridge patients waiting for liver transplant [46]. The development of BAL systems for short-term liver support must incorporate a functional cell source. As we were able to cultivate functional primary human hepatocytes for long term, our tissue engineering approach of culturing primary human cells within the native liver ECM could be adapted to further implement current liver support devices.

Finally, our hydraulic system could be easily upgraded into an automated circuit, as already described in other devices [47,48]. A reservoir connected to a pump controlled by a microcontroller-based unit, would provide automatic filling of the chamber with media followed by emptying and recirculation, and sampling of aliquots for analysis.

In conclusion, we have designed and validated a novel bioreactor for whole-liver bioengineering, showing stronger support of cell survival and metabolism compared to static cultures, longitudinal sampling and analysis of cell distribution and viability, maintenance of sterility and suitability for circulation of live cells for the development of complex 3D liver disease models.

## Data Availability

The data presented in this study are available on request from the corresponding author.

## Supplementary Materials

The following are available online at https://www.mdpi.com/2079-4991/11/2/275/s1, Table S1: CNC machine setting for the realization of the chambers; Table S2: Primary antibodies; Table S3: qPCR probes.
