# Supplementary material for: A Perfusion Bioreactor for Longitudinal Monitoring of Bioengineered Liver Constructs
Source: Nanomaterials (Basel). 2021 Jan 21;11(2):275. doi: 10.3390/nano11020275 (PMC7912543; doi:10.3390/nano11020275)
Supplement: Supplementary file 1 [file nanomaterials-11-00275-s001.pdf]

Supplementary materials

Supplementary Table S1: CNC machine setting for the realization of the chambers. The same settings were used for realization of both chambers. Two 'projects' were realized, one 3D and one 2D. ″Tool Diameter″ is the diameter of the tool used for the printing; ″Step Over″ is the spatial increase from right to left; ″Step Down″ is that of lowering; ″Feed Rate″ is the spatial movement from right to left; ″Plunge Rate″ from top to bottom; "Tolerance" and ″Allowance″ refer to the accuracy of implementation.

	**External Pathway (3D project)**		**Internal Pathway (2D project)**
	**Roughing**	**Finishing**	
**Tool diameter**	6 mm (end mill)	6 mm (end mill)	6 mm (end mill)
**Step over**	0.1 mm	0.2 mm	0.1 mm
**Step down**	1 mm	0.2 mm	1 mm
**Feed rate**	40 mm/min	40 mm/min	40 mm/min
**Plunge rate**	10 mm/min	10 mm/min	10 mm/min
**Spindle rate**	10000 rpm	10000 rpm	10000 rpm
**Tolerance**	0.1 mm	0.001 mm	0.1 mm
**Allowance**	0.5 mm	0 mm	0.5 mm

Supplementary Table S2: Primary Antibodies. List of the primary antibodies used for immunofluorescence.

**Antigen**	**Company**	**Cat.No.**	**Dilution**
Albumin	Abacm	ab106582	1/200
Cytokeratin 18	Dako	M7010	1/100
Cleaved Caspase3	CellSignaling	9661	1:100
Cytochrome 3A4	GeneTex	GTX60577	1:500
KI67	Abcam	ab15580	1:500

Supplementary Table S3: qPCR probes. List of Taqman qPCR probes used in the study (all from Integrated DNA Technologies, IDT).

**Gene Name**	**RefSeq Number**	**IDT Assay Name**
ACTB	NM_001101	Hs.PT.39a.22214847
ALB	NM_000477(1)	Hs.PT.56a.1501965
CDH1	NM_004360	Hs.PT.58.3324071
CYP1A2	NM_000761(1)	Hs.PT.58.45671878
CYP3A4	NM_017460	Hs.PT.58.1272782

FOXA2	NM_153675	Hs.PT.58.22972176
HNF4A	NM_178850	Hs.PT.58.22303533
HPRT1	NM_000194	Hs.PT.58v.45621572
MKI76	NM_002417	Hs.PT.58.27920212
SERPINA1	NM_001127702	Hs.PT.58.2013281
UGT1A1	NM_000463(1)	Hs.PT.58.40269971
